# Cortical Dopamine Transmission as Measured with the [^11^C]FLB 457 – Amphetamine PET Imaging Paradigm Is Not Influenced by COMT Genotype

**DOI:** 10.1371/journal.pone.0157867

**Published:** 2016-06-20

**Authors:** Rajesh Narendran, Divya Tumuluru, Maureen A. May, Kodavali V. Chowdari, Michael L. Himes, Kelli Fasenmyer, W. Gordon Frankle, Vishwajit L. Nimgaonkar

**Affiliations:** 1 Department of Radiology, University of Pittsburgh, Pittsburgh, Pennsylvania, 15213, United States of America; 2 Department of Psychiatry, University of Pittsburgh, Pittsburgh, Pennsylvania, 15213, United States of America; 3 Department of Human Genetics, University of Pittsburgh, Pittsburgh, Pennsylvania, 15213, United States of America; 4 Allegheny Health Network Cancer Genetics Program, Pittsburgh, Pennsylvania, 15213, United States of America; University of Manchester, UNITED KINGDOM

## Abstract

Basic investigations link a Val158Met polymorphism (rs4680) in the catechol-O-methyltransferase (COMT) gene to not only its enzymatic activity, but also to its dopaminergic tone in the prefrontal cortex. Previous PET studies have documented the relationship between COMT Val158Met polymorphism and D_1_ and D_2/3_ receptor binding potential (BP), and interpreted them in terms of dopaminergic tone. The use of baseline dopamine D_1_ and D_2/3_ receptor binding potential (BP_ND_) as a proxy for dopaminergic tone is problematic because they reflect both endogenous dopamine levels (a change in radiotracer's apparent affinity) and receptor density. In this analysis of 31 healthy controls genotyped for the Val158Met polymorphism (Val/Val, Val/Met, and Met/Met), we used amphetamine-induced displacement of [^11^C]FLB 457 as a direct measure of dopamine release. Our analysis failed to show a relationship between COMT genotype status and prefrontal cortical dopamine release. COMT genotype was also not predictive of baseline dopamine D_2/3_ receptor BP_ND_.

## Introduction

Previous studies have demonstrated that the catabolic enzyme catechol-O-methyltransferase (COMT) plays a major role in regulating dopamine transmission in the prefrontal cortex, a region that is relatively devoid of dopamine reuptake transporters, compared to the striatum [1, 2]. Human studies also link the functional polymorphism Val158Met (rs4680) in the COMT gene to changes in COMT enzymatic activity. The Met allele resulting from a G>A substitution is associated with reduced COMT enzyme activity in the brain, compared with the Val allele [3]. This has led investigators to postulate that lower and higher COMT activity, as determined by the COMT genotype in individuals, underlies increased and decreased dopaminergic tone, respectively, in the prefrontal cortex (dopamine levels in Met/Met > Met/Val > Val/Val) [4]. These data have led to studies investigating the relationship between COMT Val158Met genotype and dopamine D_1_ and D_2/3_ receptor binding potential, BP_ND_ [5, 6]. The results of these COMT genotype studies, in which baseline dopamine D_1_ and D_2/3_ receptor BP_ND_ were used as a proxy for endogenous dopamine tone, are inconsistent. In a study of 28 healthy controls with [^11^C]NNC 112 and PET, Slifstein et al., reported that Val homozygotes had higher D_1_ receptor BP compared to Met carriers and homozygotes in cortical regions. Higher D_1_ receptor BP_ND_ in the cortex of Val/Val subjects compared to Met carriers in this study was interpreted as a marker of decreased dopamine. Such an interpretation is consistent with the notion that COMT clears cortical dopamine ~40% more efficiently in individuals with the COMT Val compared to the Met allele [7]. However, in a study of 38 healthy controls with the dopamine D_2/3_ antagonist radiotracer [^11^C]FLB 457 and PET, Hirvonen and colleagues found no significant association between the Val158Met genotype and cortical D_2/3_ receptor BP_ND_. The reason for the discrepant results observed with [^11^C]NNC 112 and [^11^C]FLB 457, which are both reportedly sensitive to chronic alterations in endogenous dopamine (despite the fact that they label different subtypes of the dopamine receptor), is unclear [8, 9]. It is possible that the relatively low signal to noise ratio for [^11^C]FLB 457 in the cortex makes its baseline BP_ND_ a less than ideal proxy for endogenous dopamine tone. It is also possible that Val158Met has an effect on D_1_ receptor BP_ND_ that is independent of endogenous dopamine levels, and this is not necessarily reflected in the D_2/3_ receptor BP_ND_. The use of D_1_ and D_2/3_ receptor BP_ND_ as a proxy to interpret vivo dopamine levels in these prior studies complicates the interpretation of these data.

Recent studies with [^11^C]FLB 457 and PET have demonstrated the ability to measure dopamine transmission in the prefrontal cortex following an acute amphetamine challenge [9–11]. In human studies, amphetamine leads to a 5 to 13% reduction in [^11^C]FLB 457 BP_ND_ in several cortical regions of interest including the dorsolateral prefrontal cortex, orbital frontal cortex, medial prefrontal cortex, and anterior cingulate cortex. This reduction in [^11^C]FLB 457 BP_ND_ following an amphetamine challenge has been shown to linearly correlate with increases in extracellular fluid dopamine as measured using microdialysis in non-human primates [12]. The ability to measure amphetamine-induced displacement of [^11^C]FLB 457 with PET provides an opportunity to test the relationship between dopamine transmission and COMT Val158Met genotype status. Here, we include data from a cohort of healthy controls who underwent [^11^C]FLB 457 PET scans before and after an acute oral amphetamine challenge (0.5 mg/kg). In this dataset, the relationship between COMT Val158Met genotype status and cortical [^11^C]FLB 457 BP_ND_, as well as amphetamine-induced dopamine release (i.e., change in [^11^C]FLB 457 BP_ND_ following an acute amphetamine challenge, ΔBP_ND_), were examined. Based on the literature, we hypothesized that the COMT Val158Met genotype would be predictive of amphetamine-induced ΔBP_ND_ (Met/Met > Val/Met > Val/Val), but **not** baseline D_2/3_ receptor BP_ND_ in cortical regions.

## Materials and Methods

### Ethics statement

The Institutional Review Board and Radioactive Drug Research Committee of the University of Pittsburgh approved the study. All subjects provided written informed consent.

### Participants

Data were acquired following written informed consent in a protocol approved by both the University of Pittsburgh Institutional Review Board and the Radioactive Drug Research Committee. Amphetamine-induced displacement of [^11^C]FLB 457 BP_ND_ and COMT Val158Met genotyping (Val/Val, Val/Met, Met/Met) were available for n = 31 healthy control (8 females/23 males; 2 Asian/4 African American/25 Caucasian; see Table 1 for detailed demographics). Imaging data from 17/31 subjects were previously published [19]

**Table 1:** Demographics of the clinical sample.

	Val/Val	Val/Met	Met/Met	*p value*
N (%)	11 (35.5%)	14 (45.2%)	6 (19.3%)	
Gender (% females)	9M/2F (18%)	10M/4F (29%)	4M/2F (33%)	0.75
Ethnicity	2AA/8C/1Other	1AA/12C/1Other	1AA/5C/0Other	0.86
Smoking Status (% smokers)	7 Yes / 4 No (64%)	8 Yes / 6 No (57%)	1 Yes / 5 No (17%)	0.15
Age (years)	26 ± 4	26 ± 5	25 ± 4	0.82
Weight (kg)	76.4 ± 11.4	75.4 ± 10.4	72.1 ± 12.7	0.75

### Inclusion/Exclusion criteria

Study criteria for inclusion was 1) male or female aged 18–40 years; 2) no current or past DSM-IV axis I disorder as assessed by the Structured Clinical Interview for DSM-IV Axis I Disorders; 3) no current use of cocaine, opiates, cannabis, sedative-hypnotics, amphetamines, 3,4-methylenedioxy-N-methylamphetamine, or phencyclidine (as confirmed by urine drug screen at the study screening and PET scan day); 4) not currently pregnant or nursing; 5) no current or past chronic medical or neurological illnesses as assessed by a complete physical examination and laboratory examination; 6) no resting systolic blood pressure >140 mm Hg and no diastolic blood pressure >90 mm Hg; 7) no history of prior radioactivity exposure from nuclear medicine studies or occupation; 8) no metallic objects in the body that are contraindicated for MRI; 9) no more than one risk factor for coronary artery disease (e.g., smoking, high cholesterol, sedentary lifestyle, diabetes, hypertension, obesity, etc.); 10) no first-degree relative with a psychotic or mood disorder; and 11) not currently on any prescription or over-the-counter medications.

### Image Acquisition and Analysis

A magnetization prepared rapid gradient echo structural MRI scan was obtained using a Siemens 3 Tesla Trio scanner for determination of regions of interest. All but eight subjects underwent the baseline and post-amphetamine [^11^C]FLB 457 PET scan on the same day. In the remaining subjects, the baseline and post-amphetamine scans were acquired on different days, approximately a week apart. The details of this imaging protocol have been published [9].

Briefly, [^11^C]FLB 457 was synthesized using the methodology reported by Halldin, et al. [13]. PET imaging sessions were conducted with the ECAT EXACT HR+ camera. Following a transmission scan, subjects received an intravenous bolus injection of [^11^C]FLB 457 and emission data was collected for 90 minutes. Arterial blood samples were collected to measure the plasma free fraction (f_P_) for [^11^C]FLB 457 and derive a metabolite corrected arterial input function for modeling using methods described previously [9]. The maximum injected mass for [^11^C]FLB 457 was restricted to 0.6 μg [14]. The post-amphetamine [^11^C]FLB 457 scan was performed ~ 3 to 4.5 hours after the administration of 0.5 mg kg^-1^ of oral d-amphetamine. Amphetamine blood levels were measured before the post-amphetamine [^11^C]FLB 457 PET scan and were analyzed using methods previously described [15].

PET data were reconstructed and processed with the image analysis software MEDx (Sensor Systems, Inc., Sterling, Virginia) and SPM2 (www.fil.ion.ucl.ac.uk/spm) as described in [9]. Frame-to-frame motion correction for head movement and MR-PET image alignment were performed using a mutual information algorithm implemented in SPM2. Time activity curves were generated in MEDx for the eight cortical regions of interest and cerebellum (reference region) by using the criteria and methods previously described [9, 16]. Sampled cortical regions (n = 8) included the medial temporal lobe (MTL), dorsolateral prefrontal cortex (DLPFC), orbital frontal cortex (OFC), medial prefrontal cortex (MPFC), anterior cingulate cortex (ACC), temporal cortex (TEMP), parietal cortex (PAR), and occipital cortex (OCC). Derivation of [^11^C]FLB 457 V_T_ in the regions of interest (V_T ROI_) and cerebellum (V_ND_) was performed using a two-tissue compartment kinetic analysis using the arterial input function as previously described [9].

PET outcome variables are defined in accordance to the consensus nomenclature for *in vivo* imaging of reversibly binding radioligands [17]. D_2/3_ receptor availability at baseline and post-amphetamine was estimated using BP_ND_, i.e., binding potential relative to non-displaceable uptake, which was derived as

$$BP_{ND}=\frac{V_{T\;ROI}-V_{ND}}{V_{ND}}=f_{ND}*\;\;\frac{Bavail}{KD}$$ (1)

where, f_ND_ represents the free fraction of [^11^C]FLB 457 in the non-displaceable compartment, Bavail is the density of D_2/3_ receptors (nmol L^-1^) available to bind [^11^C]FLB 457 *in vivo*, KD is the *in vivo* equilibrium dissociation constant of [^11^C]FLB 457 (nmol L^-1^)

The amphetamine-induced change in BP_ND_ (Δ BP_ND_) was calculated as the difference between BP_ND_ measured in the post-amphetamine condition (BP_ND AMPH_) and BP_ND_ measured in the baseline condition (BP_ND BASE_), and expressed as a percentage of BP_ND BASE_

$$\Delta BP_{ND}=\;100*\frac{BP_{ND\;AMPH}-BP_{ND\;BASE}}{BP_{ND\;BASE}}$$ (2)

### COMT (Val/Met) Genotypes

DNA was extracted from frozen blood samples of subjects using the QIAMP DNA Blood Mini Kit. DNA was diluted to working stock (10ng ul^-1^). PCR primers were designed around COMT rs4680 (G/A polymorphism) using PRIMER3 program (http://bioinfo.ut.ee/primer3-0.4.0/). The size of the amplicon is 348bp. The primer sequences are F- GGGCCTACTGTGGCTACTCA and R-CTTGGCAGTTTACCCAGAGC. PCR conditions were 94° for 10 minutes, followed by 35 cycles of 94° for 30 seconds, 54° for 30 seconds 72° for 30 seconds, and final extension at 72° for 7 minutes. PCR products were checked on 1.5% agarose gel. After gel checks, PCR products were treated with ExoSAP-IT and DNA sequencing was conducted using the BigDye Terminator V3.1 Cycle Sequencing Kit (Life Technologies). Sequencing traces were compiled on Sequencher V5.3 and two investigators read genotypes traces separately to confirm them. Discrepancies were resolved through re-analysis.

### Statistical Analysis

Group wise differences in demographics were tested using Chi-squared and analysis of variance (as applicable). Comparison between baseline and post-amphetamine scan variables (injected dose, specific activity, injected mass, free fraction in plasma, clearance and cerebellum V_ND_) were performed with paired t-tests. Prior to performing statistical tests, Box's M tests were performed on the dependent variables, BP_ND_ and ΔBP_ND_, to evaluate the data, where p >0.05. Thus, genotype effect on baseline cortical BP_ND_ and Δ BP_ND_ was tested using a linear mixed model analysis with cortical regions of interest as a repeated measure and genotype as the fixed factor (IBM SPSS Statistics). Genotype, region, and genotype by region interaction were included in the model as explanatory variables. To control for the potential confounding effect of smoking status, its effect on the model was evaluated by including it as a factor following the primary analysis. A two-tailed probability value of p < 0.05 was selected as the significance level for all analyses.

## Results

### Demographic and scan parameters

Genotype frequencies, age, sex, ethnicity, smoking status and weight are included in Table 1. The genotype distribution is consistent with Hardy Weinberg expectations. The allele frequency in the sample was consistent with estimates among individuals with Caucasian ancestry [18]. No significant differences were noted in any of these variables (smoking status was at p = 0.15, however, the reliability of this test was low due to there being only one smoker in the Met/Met group). Table 2 shows no significant differences in the baseline and post-amphetamine [^11^C]FLB 457 scan parameters in all three genotype groups. No significant differences were noted in the genotyped groups in the amphetamine blood levels measured at time, t = 0 min relative to post-amphetamine [^11^C]FLB 457 scan (**data in** Table 2).

**Table 2:** [^11^C]FLB 457 baseline and post-amphetamine scan parameters.

	Val/Val		Val/Met		Met/Met	
	(n = 11)		(n = 14)		(n = 6)	
	Baseline	Amphetamine	Baseline	Amphetamine	Baseline	Amphetamine
Injected Dose (mCi)	7.4 ± 1.5	8.1 ± 0.6	7.8 ± 1.3	7.6 ± 1.3	7.6 ± 1.6	7.0 ± 2.4
Specific Activity (Ci mmol^-1^)	10752 ± 6754	9639 ± 3764	9925 ± 4753	8245 ± 4184	10863 ± 8124	8290 ± 6983
Injected Mass (μg)	0.4 ± 0.2	0.4 ± 0.1	0.4 ± 0.1	0.4 ± 0.1	0.4 ± 0.2	0.4 ± 0.2
Free Fraction in Plasma (%)	36.3% ± 7.5%	35.0% ± 6.4%	38.4% ± 7.3%	37.6% ± 6.0%	38.9% ± 4.6%	35.8% ± 9.5%
Clearance (l h^-1^)	75 ± 19	72 ± 29	71 ± 28	74 ± 27	79 ± 14	84 ± 19
Cerebellum V_ND_ (mL cm^-3^)	4.17 ± 0.69	3.94 ± 0.54	4.65 ± 1.31	4.47 ± 1.07	4.77 ± 0.98	4.30 ± 0.74
Amphetamine Level (ng mL^-1^)	-	79.0 ± 12.5^1^	-	76.5 ± 13.8	-	75.3 ± 8.6^2^

V_ND_ Volume of distribution for nondispaceable tissue uptake

^1^ Amphetamine samples only available for n = 10 out of 11 subjects

^2^ Amphetamine samples only available for n = 5 out of 6 subjects

### D_2/3_ receptor availability (BP_ND_) under baseline conditions

**Fig 1:**
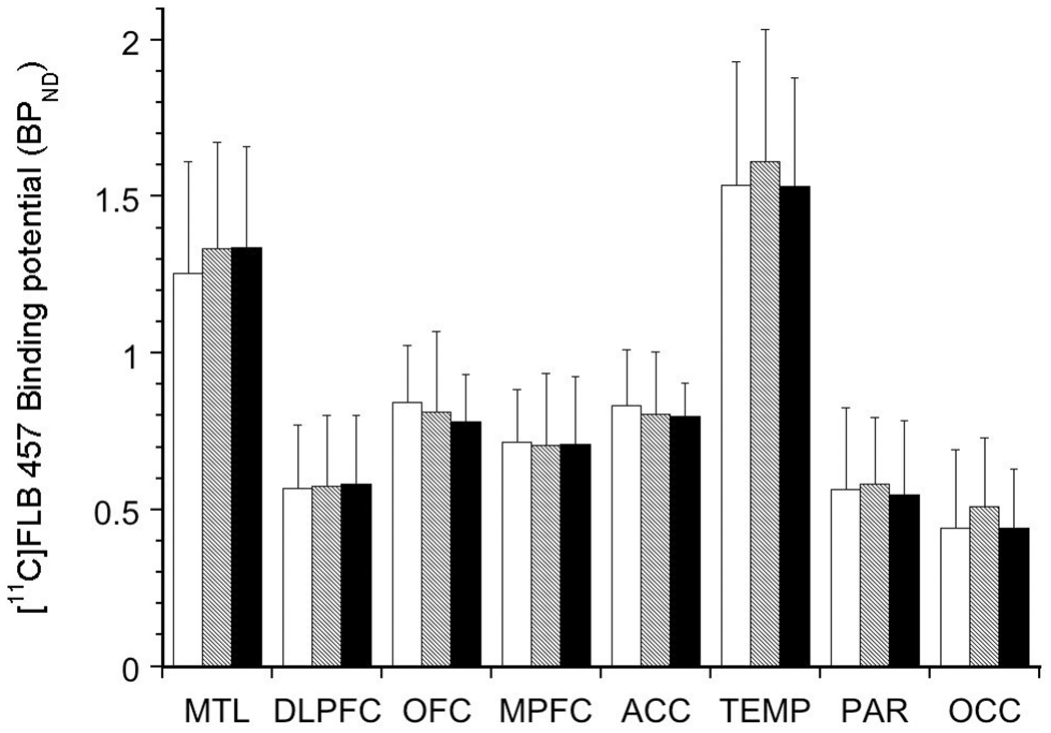
Baseline [^11^C]FLB 457 Binding Potential (BP_ND_) in Genotyped Healthy Subjects. The bar graph shows the lack of difference in D_2/3_ receptor availability in cortical regions of interest comparing Val/Val (white bars), Val/Met (shaded bars), and Met/Met (black bars) genotyped healthy subjects.

As shown in Fig 1, no differences in baseline [^11^C]FLB 457 BP_ND_ were observed between the genotypes (linear mixed model, effect of genotype, F (2, 28) = 0.04, p = 0.96; effect of region, F (7, 196) = 186.96, p < 0.001; genotype x region interaction, F (14, 196) = 0.39, p = 0.98). The inclusion of smoking as a factor in the model did not alter the results (genotype, p = 0.96; genotype* region interaction, p = 0.85). BP_ND_ derived using a simplified reference tissue method with the cerebellum as an input function did not alter the results (genotype, p = 0.94; region, p <0.001; genotype*region interaction, p = 0.94)

### Amphetamine-induced reduction in D_2/3_ receptor availability (Δ BP_ND_)

**Fig 2:**
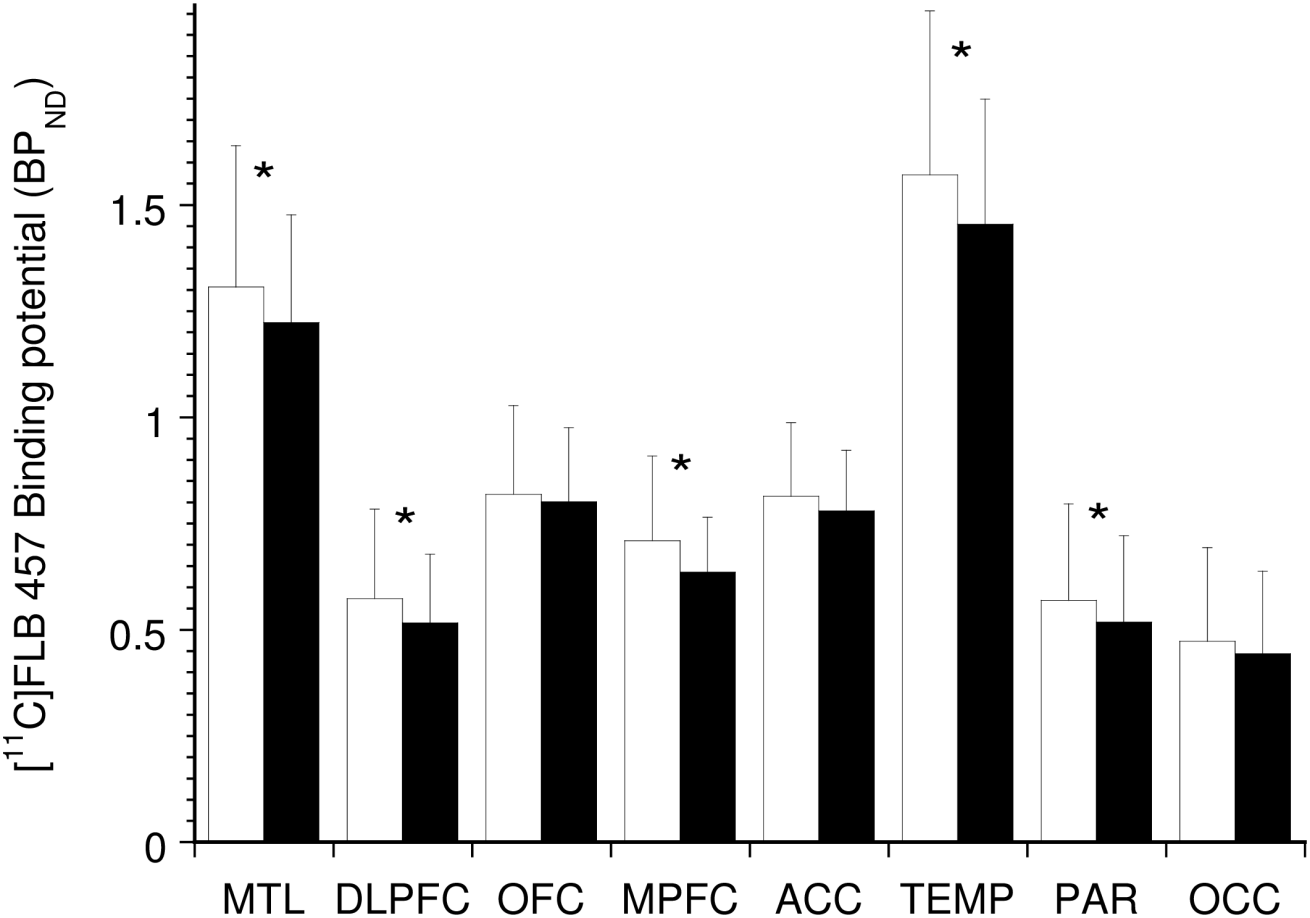
Amphetamine-Induced Displacement of [^11^C]FLB 457 Binding Potential. The bar graph shows [^11^C]FLB 457 BP_ND_ under baseline (white bars) and post-amphetamine (black bars) conditions in n = 31 healthy subjects. Amphetamine led to a significant decrease in five of the eight cortical regions of interest (* denotes p < 0.05, paired t-tests) consistent with previous reports [9–11].

**Fig 3:**
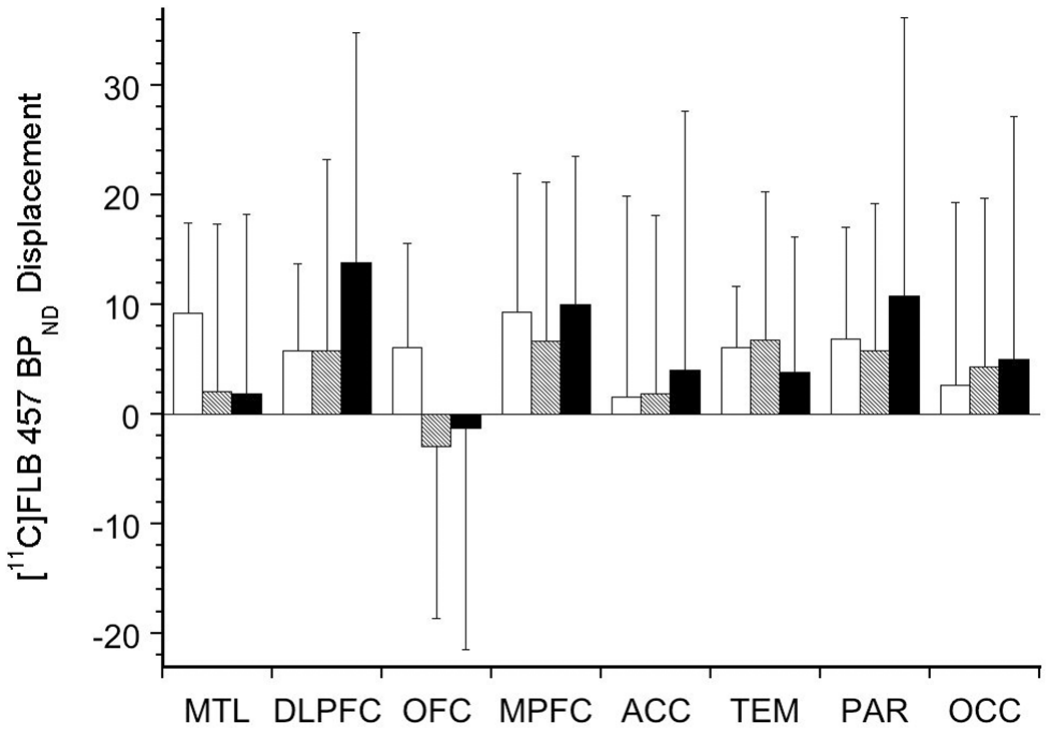
Amphetamine-Induced Δ[^11^C]FLB457 BP_ND_ in Genotyped Healthy Subjects. The bar graph shows the amphetamine-induced reduction of [^11^C]FLB 457 BP_ND_ in cortical regions of interest comparing Val/Val (white bars), Val/Met (shaded bars), and Met/Met (black bars) genotyped healthy subjects.

Consistent with previous studies [9–11, 19], amphetamine led to a significant reduction in [^11^C]FLB 457 BP_ND_ in five of the eight cortical regions of interest when data from all three genotypes were included (Fig 2). No significant differences in amphetamine-induced Δ [^11^C]FLB 457 BP_ND_ were observed between the genotypes (data shown in Fig 3; linear mixed model, effect of genotype, F (2, 28) = 0.12, p = 0.89; effect of region, F (7, 196) = 2.70, p = 0.01; genotype x region interaction, F (14, 196) = 0.96, p = 0.50). The inclusion of smoking as a factor in the model did not alter these results (genotype, p = 0.71; genotype* region interaction, p = 0.49). The use of alternative PET outcome measures such as ΔV_T_ and ΔBP_P_ showed no significant effect for genotype consistent with that observed with ΔBP_ND_. (data not shown). ΔBP_ND_ derived using the simplified reference tissue method did not alter the results (genotype, p = 0.55; region, p = 0.03; genotype*region interaction, p = 0.62).

## Discussion

The results of this study demonstrate the COMT Val158Met genotype is predictive of neither D_2/3_ baseline receptor BP_ND_ nor amphetamine-induced ΔBP_ND_ in the cortical regions. The failure to demonstrate a relationship between COMT Val158Met genotype and cortical D_2/3_ receptor BP_ND_, which is consistent with a previously published report, was expected [6]. However, the failure to demonstrate a connection between COMT Val158Met genotype and cortical dopamine transmission is surprising given that basic studies support such a relationship [7]. This is also inconsistent with the numerous human studies that link COMT genotype with prefrontal cortical dopamine-dependent behaviors such as cognition, aggression and impulsivity [20–22]. The exact reasons that led to an inability to link COMT Val158Met genotype with amphetamine-induced displacement of [^11^C]FLB 457 are unclear. One factor could be the relatively high inter-individual variability associated with ΔBP_ND_. This is likely due to the relatively lower baseline D_2/3_ BP_ND_ and decreased dopamine released in response to amphetamine in the cortex compared to that in the striatum [12]. This issue is highlighted in the literature by studies that have failed to displace the vivo binding of [^11^C]FLB 457 in the cortex following an acute amphetamine challenge [23, 24]. Here, this is evident in Fig 3, where the standard deviations for ΔBP_ND_ for the three different genotype groups in the cortical regions are high. For example, in the dorsolateral prefrontal cortex, the amphetamine-induced ΔBP_ND_ in Met/Met (-14 ± 21%) individuals is numerically, but ***not statistically***, greater than that in Val/Val (-6 ± 8%) and Val/Met (-6 ± 18%) individuals. However, to demonstrate whether this numerical difference reaches statistical significance in Met/Met compared to Val/Val and Val/Met, it would require [^11^C]FLB 457-amphetamine data in 128 and 200 subjects respectively, as indicated by power calculations (β = 0.8). The heterogeneity of the subject population (e.g., large age range, gender, smoking status etc.,) and differences in scanning schedule (acquisition of baseline and post-amphetamine scans on the same vs. different day) may have also contributed to the variable Δ in BP_ND_. Another factor that may have contributed to the inability to demonstrate an effect for COMT genotype on ΔBP_ND_ in our paradigm is the fact that the half-life for amphetamine-induced dopamine release is relatively prolonged in the cortex [25]. Studies have shown that the half-life (t_1/2_) for dopamine released in the cortex is ~ 2.5 hours for intravenous amphetamine, which peaks immediately (< 5 minutes) in the plasma. This is relevant because the timing of the [^11^C]FLB 457 post-amphetamine scan (i.e., three hours after the oral amphetamine administration) in our studies was designed to capture the peak dopamine release that follows the peak amphetamine plasma level (~ 3–4 hours), and not necessarily the clearance, which is measured following several half-lives. [^11^C]FLB 457 PET studies measuring time to restore baseline BP_ND_ rather than the magnitude Δ in BP_ND_ following an acute amphetamine challenge might be more relevant in detecting a difference in the clearance of dopamine as influenced by COMT Val158Met genotypes. It is also possible that alpha-methyl-para-tyrosine (AMPT) induced dopamine depletion PET studies that measure baseline dopamine levels may be more successful in detecting an effect for the COMT genotype. However, measuring baseline dopamine levels in the cortex with such an imaging paradigm has been unsuccessful due to technical challenges [26, 27]. Consistent with this is the notion that the COMT genotype influences tonic more than phasic dopamine levels [28]. Such a line of reasoning would explain the D_1_ receptor findings reported with COMT genotype because D_1_ receptors that are located in the extra synaptic space likely reflect slower adaptations, including changes in gene transcription in response to chronically altered tonic dopamine levels. It would also explain the lack of a relationship between COMT genotype and amphetamine-induced dopamine release, which is likely more reflective of the phasic DA release caused by behaviors.

To summarize, the strengths of this study include the use of a well-validated imaging paradigm to measure dopamine release in the cortex and inclusion of a reasonable sample size by PET study standards. The weaknesses of the study include increased variability associated with the dopamine release outcome measure ΔBP_ND_ and questions related to how well the amphetamine-induced dopamine release paradigm captures the effect of the COMT genotypes on dopamine clearance. In conclusion, we were not able to demonstrate an effect for the COMT Val158Met genotype on either cortical D_2/3_ receptor BP_ND_ or amphetamine-induced dopamine transmission.
